# Analyzing the locomotory gaitprint of *Caenorhabditis elegans* on the basis of empirical mode decomposition

**DOI:** 10.1371/journal.pone.0181469

**Published:** 2017-07-24

**Authors:** Li-Chun Lin, Han-Sheng Chuang

**Affiliations:** 1 Department of Biomedical Engineering, National Cheng Kung University, Tainan, Taiwan; 2 Medical Device Innovation Center, National Cheng Kung University, Tainan, Taiwan; INSERM U869, FRANCE

## Abstract

The locomotory gait analysis of the microswimmer, *Caenorhabditis elegans*, is a commonly adopted approach for strain recognition and examination of phenotypic defects. Gait is also a visible behavioral expression of worms under external stimuli. This study developed an adaptive data analysis method based on empirical mode decomposition (EMD) to reveal the biological cues behind intricate motion. The method was used to classify the strains of worms according to their gaitprints (i.e., phenotypic traits of locomotion). First, a norm of the locomotory pattern was created from the worm of interest. The body curvature of the worm was decomposed into four intrinsic mode functions (IMFs). A radar chart showing correlations between the predefined database and measured worm was then obtained by dividing each IMF into three parts, namely, head, mid-body, and tail. A comprehensive resemblance score was estimated after k-means clustering. Simulated data that use sinusoidal waves were generated to assess the feasibility of the algorithm. Results suggested that temporal frequency is the major factor in the process. In practice, five worm strains, including wild-type N2, TJ356 (*zIs356*), CL2070 (*dvIs70*), CB0061 (*dpy-5*), and CL2120 (*dvIs14*), were investigated. The overall classification accuracy of the gaitprint analyses of all the strains reached nearly 89%. The method can also be extended to classify some motor neuron-related locomotory defects of *C*. *elegans* in the same fashion.

## Introduction

*Caenorhabditis elegans* is a popular multicellular model animal used to explore neural circuits, behavior, and genes at system level [1]. *C*. *elegans* was introduced to the community by Sydney Brenner in 1978 [2]. Since then, tremendous worm-based research focusing on neuroscience, genetic engineering, and environmental toxicology has been conducted [3–5]. In 1986, a map of all the 302 neurons in the *C*. *elegans* nervous system and the 7,000 connections or synapses among these neurons was first published [6]. The complete genome sequence was established and revealed to have more than 60% genetic similarity with humans [7]. Subsequently, many researchers have been dedicated to bridge the gaps among specific genes, neural circuits, and behavioral phenotypes. Genes that are mediating the worm locomotion are frequently investigated [8]. In addition, investigating how perturbation in the neural network triggers the changes in locomotion is of interest to researchers [9]. Understanding the mechanism of gait switching of *C*. *elegans* has important implications to motor-neurological diseases in humans [10]. However, *C*. *elegans* performs diverse behavioral patterns even with limited neurons. Thus, studying locomotion in relation to genes and neural activities remains a big challenge until today [11].

Despite these difficulties, numerous researchers remain committed to the deciphering of the codes behind the locomotory gaits exerted by *C*. *elegans*. In contrast to genes and neurons, gaits are visible to human eyes. Previous studies have thoroughly observed and then characterized the *C*. *elegans* [12]. However, their results were dependent on observation, and thus, were likely to have human errors and biases. Quantitative analysis of locomotory gaits enables a systematic approach to classify the differences between worms. Distinguishing the motion changes that are linked to genetic or neural defects of a worm relies on cautious motion analysis. Biomechanical parameters, such as curvature, velocity, and frequency, are calculated from videos of worms in motion [13], from which corresponding analytical models of motion can be generated [14]. Yemini et al. reported an online behavioral database for 305 worm strains [15]. Other biomechanical parameters, such as propulsive forces and power, require a micro particle image velocimetry system [16], a microfluidic chip-based system [17, 18], or an image-based system [19]. Some studies developed algorithms to automatically detect predefined behaviors, such as omega bends and reversals [20].

Unsupervised learning for locomotion classification is another approach that uses the inherent structure of a dataset for the classification of informative patterns. Stephens et al. showed that the space of shapes adopted by *C*. *elegans* can be formulated with only four dimensions [21]. Another study used four eigenworms to describe the locomotion of the worm and built a dictionary of repetitive behavior motifs to divide worms with different genes into functional classes [22]. Recently, a study has proposed a method that has no definition of animal-specific features [23]. Histograms of commonly observed scale-invariant feature transform (SIFT) features representing nematode motility were constructed using SIFT as an elementary image feature [24].

In this study, a novel gait analysis method is presented to classify worms from strain types. The present method used the body curvature of *C*. *elegans* to classify the locomotory gaits of a worm through 2D empirical mode decomposition (2D EMD) [25] and a correlation algorithm. Unsupervised method k-means clustering was then used to classify worms according to their gait features. Resemblance scores were also calculated from the k-means clustering results to provide similarity estimation between an unknown worm and predefined database.

## Methods and materials

### Concept of gaitprint analysis

**Fig 1:**
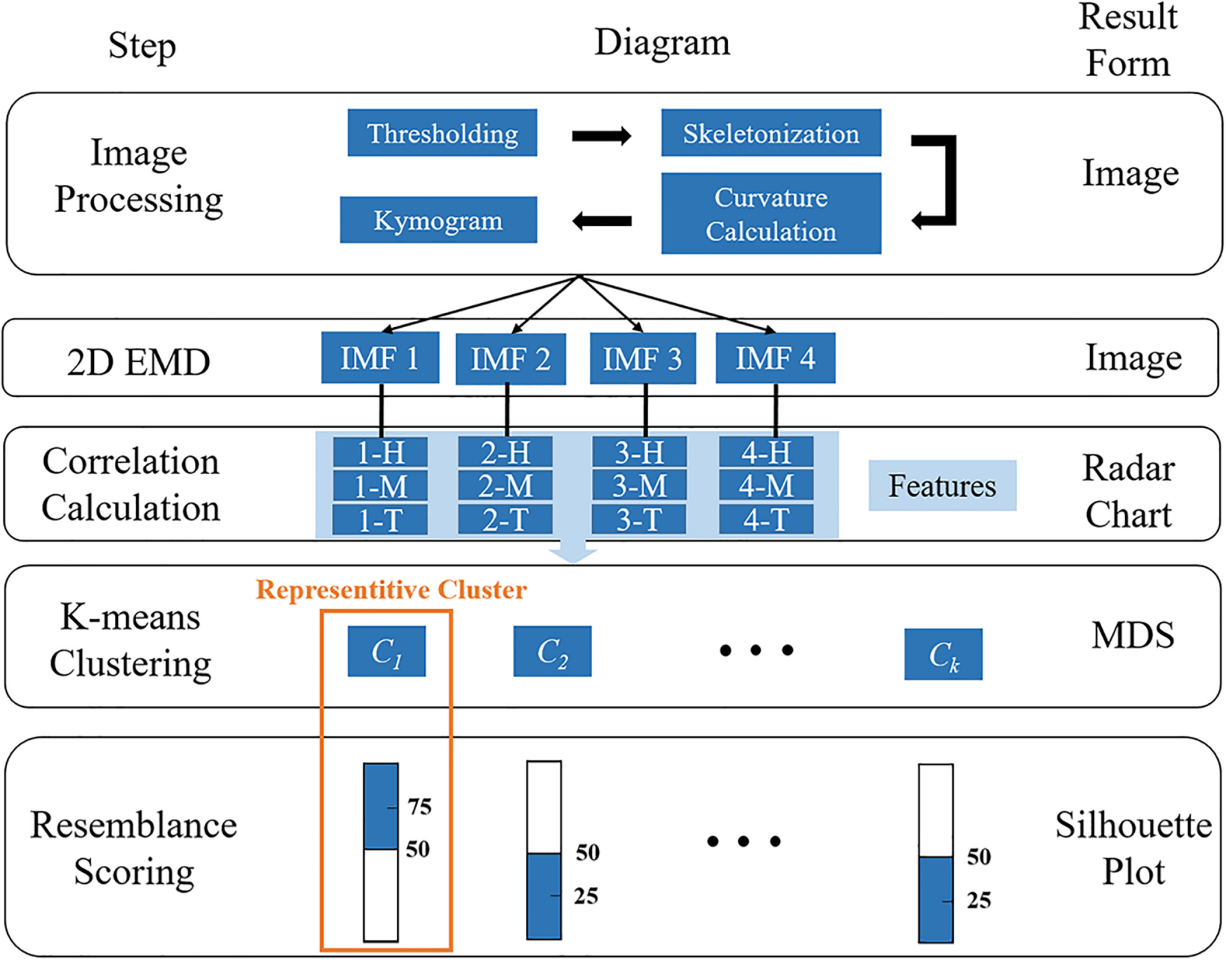
Executive flow chart of the gaitprint analysis method.

Unlike humans, *C*. *elegans* is a simple life form that possesses only few discernible signatures. However, neuron-mediated locomotion is a visible trait linked to their identities and response to environmental stimuli. Thus, this study aimed to develop an image-based algorithm named gaitprint to translate the gait patterns of a worm into a human understandable language. A recording time period of 2.5 s, which was sufficient to include at least three swimming cycles of all strains, was required in a video clip. Five steps were proposed for gaitprint analysis (Fig 1). In step 1, a worm’s body is skeletonized and then divided into 10 segments. A kymogram, containing the information of the body curvature of a worm over time, is generated. Parameters including curvature amplitude, body bend, and wavenumber are included in this 2D graph. In step 2, the kymogram is decomposed into four intrinsic mode functions (IMFs) through the 2D EMD method. These parameters are then extracted into the IMFs. In step 3, each IMF is divided into three parts, namely, head, mid-body, and tail. Consequently, 12 features are analyzed. A radar chart can be plotted by comparing the correlations between an unknown worm and predefined database in each feature. The radar chart is dubbed as a gaitprint, because each worm strain has its unique pattern. Thus far, a particular worm pattern can be recognized. However, two more steps are required if a specific number showing the comprehensive similarity between the unknown worm and predefined database is preferred. In step 4, k-means clustering is performed to quantitatively distinguish the similarities among the subjects over the 12 features. In the final step, the clustering results are calculated to yield a comprehensive resemblance score to indicate the relationship between the unknown and predefined worms. The score provides a probability instead of a specific answer. A high score (>50%) typically represents a high probability that an unknown worm and the predefined worm may belong to the same strain. Considering a wide variety of strains, the resemblance score is appropriate to describe the possible strain type of an unknown worm in this study. However, this analytical method works only for worms that express their genetic defects in locomotory phenotypes.

### Preparation of microchip

Each worm was confined in a chamber filled with the nematode growth medium (NGM) buffer on a polydimethylsiloxane (PDMS; Sylgard 184, Dow Corning) microchip. The microchip was composed of a 3 ×3 chamber array, each measuring 91 μm in height and 2 mm in diameter. A negative photoresist, SU8-2025 (Microchem), was spin-coated on a 4” silicon wafer and patterned with conventional photolithography to form the mold structure. The PDMS prepolymer was prepared by mixing the monomer Sylgard 184 with the cure agent at a volumetric ratio of 10:1. The resulting mixture was then poured onto the silicon wafer inside the glass dish for molding. The prepolymer was baked in an oven at 80°C for 1 h, and then the chamber array was obtained by peeling it off the mold. Subsequently, each chamber that is occupied by a single worm was filled with 1.5 μL of NGM buffer. A microchip for worm image recording was completed after a cover glass was placed on the PDMS.

### Worm image recording

**Fig 2:**
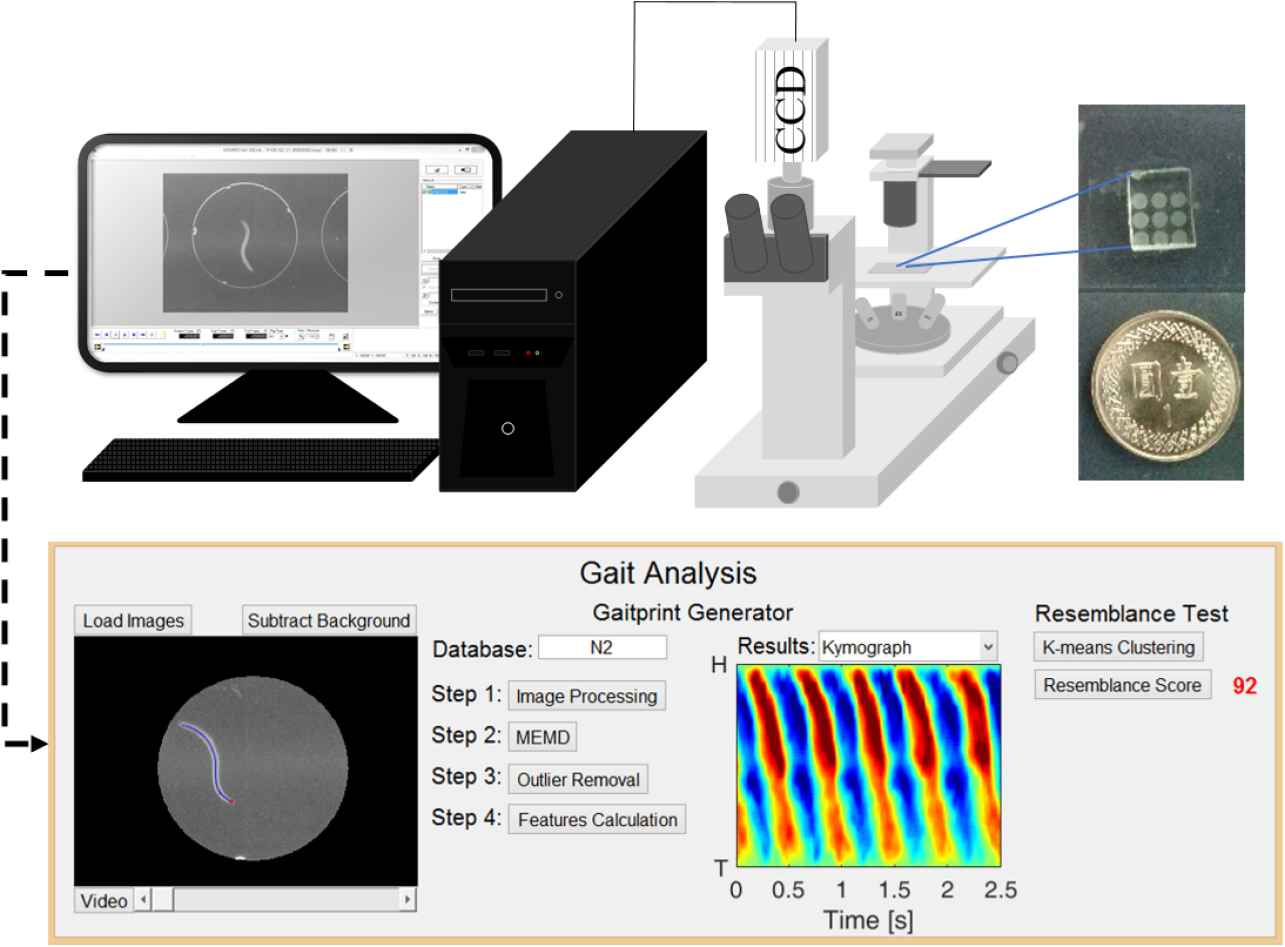
Schematic of the measurement system. Experimental setup comprising a microscope for imaging, a high speed camera, and a computer. The inset represents the actual PDMS microchip with a 3-by-3 well array. A MATLAB program was developed to automate the gaitprint analysis.

A high-speed camera (Memrecam GX-3, NAC Image Technology) was used to capture a single worm locomotion video. The camera was installed under an inverted microscope (CKX41, Olympus) coupled with a 4× magnification objective (Fig 2). A phase contrast slider (Ph2, IX2-SL) was mounted onto the microscope to enhance the visibility of the worm images. At least three swimming cycles were performed in an appropriate kymogram to avoid bias. Each worm was confined accordingly in a chamber of the microchip at room temperature of 24°C and then recorded for 2.5 s. This fixed recording time was determined because it was sufficiently long to meet the minimum requirement of all the strains used in this study. A short duration of 30–60 s was typically required for worms to resume their locomotion after they were transferred from the incubator to a microchip. Measurement was conducted after the worms showed constant and stable swimming. Although the TJ356 and CL2070 worms carrying the right-roller allele of *rol-6* moved their bodies differently from their wild-type counterpart, cyclic motion was still observed under a dissecting microscope. Therefore, repeated patterns were shown on their kymograms. The acquired image size was 640 × 480 pixels, and the recording frame rate was 100 Hz. Two strains, such as N2 and CL2070, were measured to establish their gaitprint databases. Twenty young adult worms were measured and analyzed for each strain.

### Worm culture

**Fig 3:**
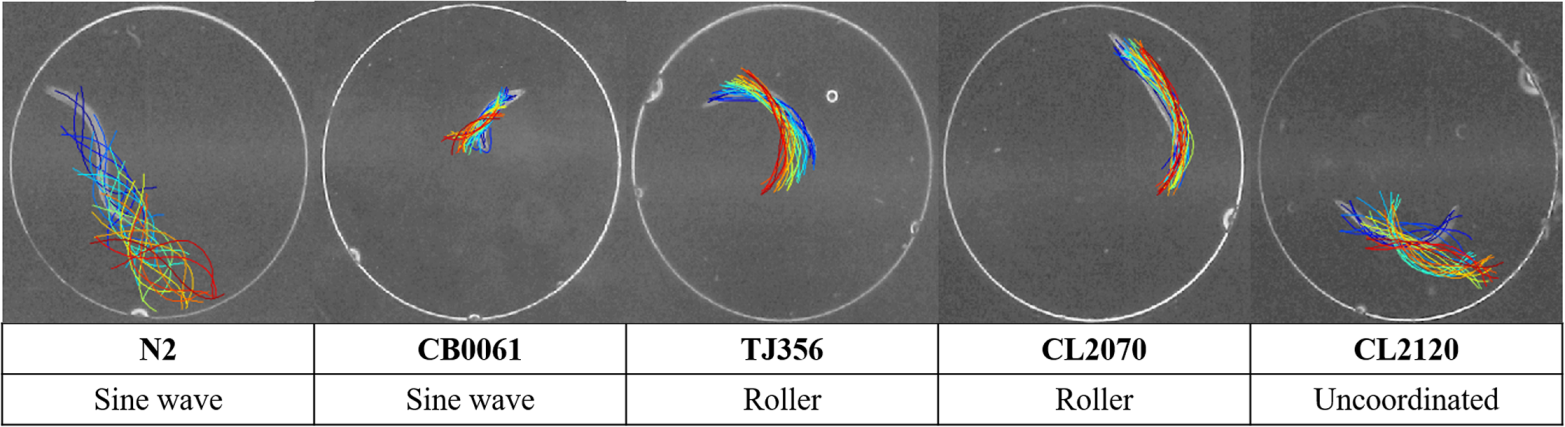
Trajectories of five worm strains used in this study. The overlapped colorful lines represent the swimming trajectories of each worm at different time points. Three types of locomotion are classified and shown in correspondence to their strains.

Wild-type *C*. *elegans*, such as N2 and four transgenic strains, were used in this research to assess the proposed gaitprint analytical method (Fig 3). These worms represented three common types of locomotion, such as sinusoidal wave, roller, and uncoordinated body movement (see S1 File and S1 Movie). Transgenic strains were obtained from Caenorhabditis Genetics Center at the University of Minnesota. All the worms were maintained on NGM agar plates seeded with *E*. *coli* as food sources and cultured by following the protocol described by Sydney Brenner [2].

The four transgenic strains used in this study were of different phenotypic behaviors. TJ356 (*zIs356*) [*daf-16*p::*daf-16*a/b::GFP + *rol-6* (*su1006*)] and CL2070 (*dvIs70*) [*hsp-16*.*2*p::GFP + *rol-6* (*su1006*)] are both rollers carrying the dominant *rol-6* allele. *Rol-6* primarily mediates the organismal morphology and produces a roller phenotype, which twists the body of the worm into a right-handed helix [26, 27]. CB0061 [*dpy-5*] has a dumpy phenotype, which is shorter and stouter than its wild type counterpart at the same developmental stage [28]. CL2120 (*dvIs14*) [(*pCL12*) *unc-54*::beta 1–42 + (*pCL26*) *mtl-2*::GFP] is an Alzheimer model animal carrying human Aβ 1–42 peptides in its muscles [29]. This strain expresses progressive and age-dependent paralysis after onset by temperature. Aβ toxicity is released when the worms are cultured at a temperature higher than 15°C. Therefore, all worms, except the CL2120 strain, were cultured in an incubator at 20°C. CL2120 strain was separately cultured at 15°C.

The worm locomotion in this study was measured on adult day 1 (AD1) at room temperature of 24°C. Bleaching was performed to obtain a synchronous population of L1 stage worms [30]. After 48 h from bleaching, L4 stage worms were transferred to a new agar plate with a platinum wire and then cultured for 24 h before measurement.

### Kymogram and two-dimensional empirical mode decomposition

**Fig 4:**
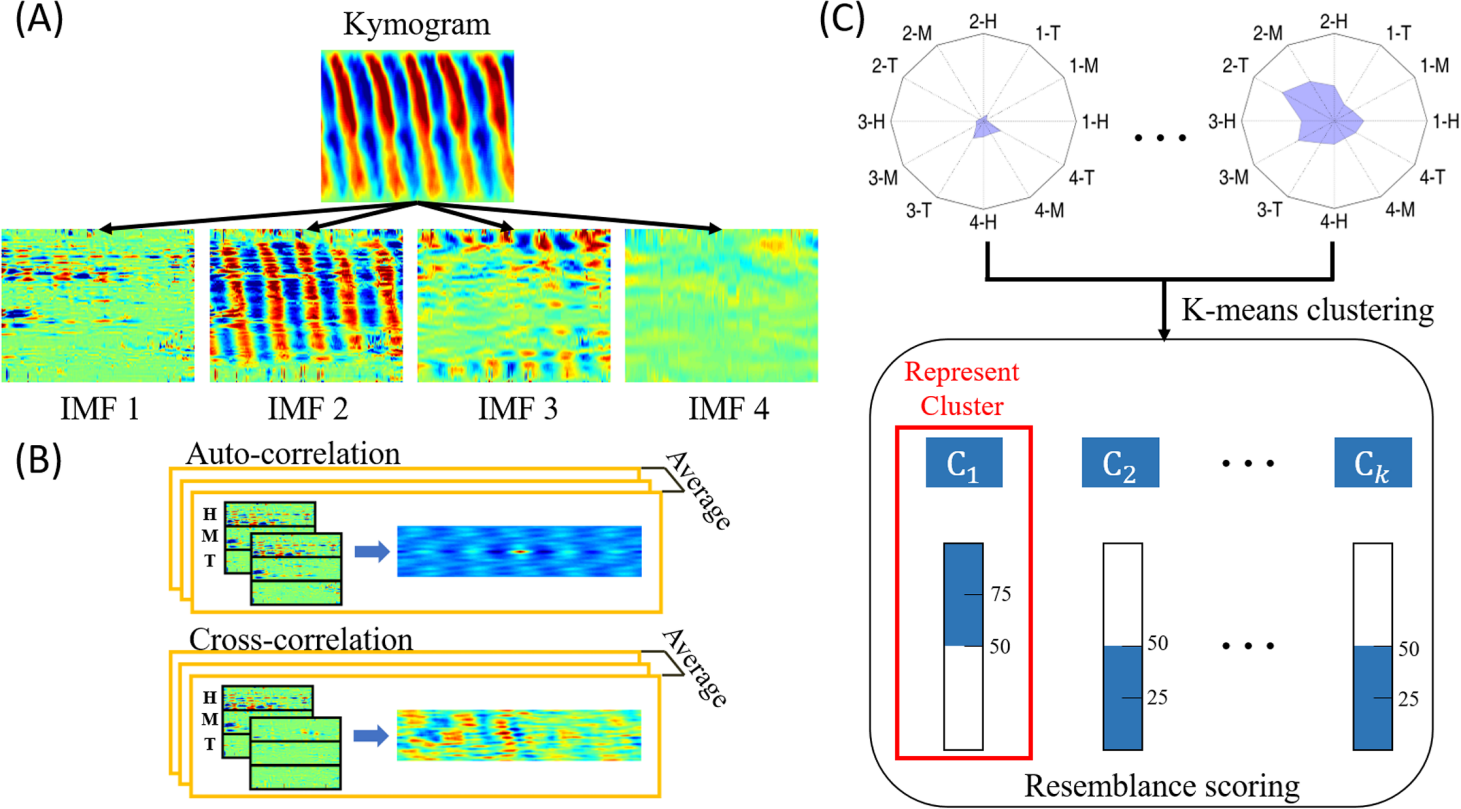
Schematic of the gaitprint analysis. (A) IMFs derived from a kymogram using the 2D EMD method. (B) Comparison of kymograms at each body part using auto-correlation and cross-correlation algorithm. (C) K-means clustering was used to classy the worms into k clusters. Resemblance scores for each worm are calculated according to the cluster it belongs.

For simplicity, each worm was represented by its body centerline. The body curvature of a worm was then derived from the centerline. Finally, a kymogram showing the relationships between the body curvature change of the worm and elapsed time was plotted. Kymogram is a 2D spatio-temporal graph containing unique information on the locomotory gait of a worm. The gait pattern may change corresponding to the worm strain and motor neuron defects. A powerful algorithm named EMD was used to extract the information from the worm locomotion. EMD was first developed by N.E. Huang in 1998 [31] and has been widely applied in signal processing fields until today. The hidden characteristics can be revealed by decomposing the original signal into a combination of IMFs through a sifting process [32]. 2D EMD was performed to classify worm strains on the basis that the body centerline of a worm was treated as a finite waveform. Kymogram was used as the original signal source in the present study to obtain 2D IMFs based on the 2D EMD (Fig 4A). The present preliminary investigation showed that the intensity of IMFs dropped below 1% after four decompositions. Therefore, only the first four IMFs were selected for subsequent analysis in this study. In principle, the later IMFs (e.g., the fourth IMF) indicated lower body bends in locomotion than the former ones (e.g., the third IMF).

### Correlation coefficients

Correlation coefficients were used to determine (1) the outliers of measured worms in a predefined database and (2) body part similarities of the kymograms and IMFs (Fig 4B). 2D fast Fourier transform was used to calculate the image-based auto-correlation and cross-correlation coefficients. The correlation peak in the 2D correlation matrix was then used to stand for the correlation degree of two images. The normalized number, which is the ratio of the cross-correlation to the auto-correlation, was then derived for subsequent gaitprint analysis.

For the first part, a large number of worms were collected in the predefined database. The eligibility of each worm was then examined by correlating its kymogram with those of the other worms of the same type to determine the outliers among the measured worms. When more than half of the correlation peak values of a worm were out of one standard deviation of the overall correlation coefficients, the worm was discarded; otherwise, the worm was kept. After the outliers were removed, worms of the same type were categorized into a predefined database. In the study, 20 worms were initially measured for each database. After the screening, four and six outliers were removed from the N2 and CL2070 databases, respectively. For the classification of an unknown worm, the 2D IMF of a worm was compared with that of a predefined database. Given that each worm generated four IMFs and each 2D IMF of a worm was divided into three parts, namely, head (H), mid-body (M), and tail (T), a total of 12 features can be used for comparison. After calculating the correlation coefficients of the 12 features by comparing with all the worms in the database, a radar chart showing the similarities of the 12 features between the unknown worm and predefined database can be plotted (Fig 4C). Every worm strain evidently has a unique radar chart associated with its locomotory gait. Therefore, the radar chart was considered as a gaitprint of the worm. The identity of an unknown worm can be determined eventually by comparing the gaitprint of an unknown worm and that of a predefined database.

### K-means clustering and resemblance scoring

Gaitprint analysis relies on an unsupervised learning method (k-means clustering) without predefining the features of worms in the database. K-means clustering is a type of classification that partitions *n* observations into *k* clusters such that each observation is included in the cluster with the nearest mean. The distance between the observation and cluster center is determined by the Euclidean distance. Worms were clustered depending on the 12 features through k-means clustering.

The consistency within data clusters can be validated through the Silhouette method [33]. A Silhouette value *s*(*i*) that is close to 1 indicates that the sample is away from the neighboring clusters. Conversely, *s*(*i*) = 0 indicates that the sample is close to the boundary between two neighboring clusters. A negative Silhouette value indicates that the sample might be assigned to the wrong cluster. The Silhouette plot of the entire clustering data shows a relative quality of the clustering and a map of the data network. In the present study, the Silhouette method was performed to determine a resemblance score of an unknown worm against a predefined database. A predefined strain was assigned to be the representative database. When a measured worm was in the same cluster with the strain, its resemblance score was $50+(\frac{s(i)-(-1)}{2})\times 50$. Otherwise, the resemblance score was $\frac{-s(i)-(-1)}{2}\times 50$. Therefore, if the measured worm was in the representative cluster and apart from the neighboring clusters (i.e., >75%), the worm was considered more likely to be a predefined strain. On the contrary, if the worm was not in the representative cluster and apart from the neighboring clusters (i.e., <25%), the worm was considered less likely to be of the same type of the predefined strain. The identity of the worm could be eventually recognized by repeating the procedure to calculate the resemblance scores of an unknown worm against predefined databases.

## Results and discussion

### Simulated models

*C*. *elegans* has muscles organized as longitudinal strips in each of the four body quadrants [34]. Wild-type *C*. *elegans* moves by alternately coordinated contraction and relaxation of the opposing dorsal and ventral muscle strips attached to the cuticle along the body length, producing sinusoidal waveforms that propel the animal forward [35]. Therefore, the locomotory gait of *C*. *elegans* was modeled using a sinusoidal traveling wave to assess the feasibility of gaitprint analysis in this study.

The locomotion of *C*. *elegans* was modeled by *Asin*(*kx* − *ωt*)*e*^−*x/l*^, where *A* is the curvature amplitude, *k* is the wavenumber (*k* = 2*π*/*λ*, *λ* is the nematode wavelength), *ω* is the angular body bend of the nematode (*ω* = 2*πf*, where *f* is the frequency in Hz), *x* represents a vector of spatial coordinates (200 pixels long), and *l* is the length of the nematode from head to tail [36].

Six simulated models (Table 1) were created by following the study of Koren et al. [23] to assess the gaitprint analysis method in the present study. Three model types (I, II, and III) were created by setting variables in curvature amplitude, frequency, and wavenumber to investigate the significance of the parameters in the EMD. Two special models (IV and V) were created by tuning the three parameters to simulate the swimming gaits of N2 and CB0061. The parameters of the two strains were referred to the experimental data. The last model (VI) was an actual wide-type example obtained from prior literature [36]. The example provided a comparison for validation of the method.

**Table 1:** Parameters of the simulated datasets.

Simulated model	Curvature amplitude, A [pixel]	Body bend, f [Hz]	Wavenumber, k [pixel^-1^]
**I**	15, 21, 30*	**2.1**	**0.035**
**II**	**21**	1.64, 2.1, 3.2*	**0.035**
**III**	**21**	**2.1**	0.0305, 0.04, 0.05*
**IV (N2)**	**25**	**2.1**	**0.035**
**V (CB0061)**	**21**	**1.64**	**0.04**
**VI [33]**	**25**	**2**	**0.035**

* W1, W2, and W3 denote simulated worms in model I that differ in curvature amplitude but the same in body bend and wavenumber. W4, W5, and W6 denote simulated worms in model II that differ in body bend but the same in curvature amplitude and wavenumber. W7, W8, and W9 denote simulated worms in model III that differ in wavenumber but the same in curvature amplitude and body bend.

**Fig 5:**
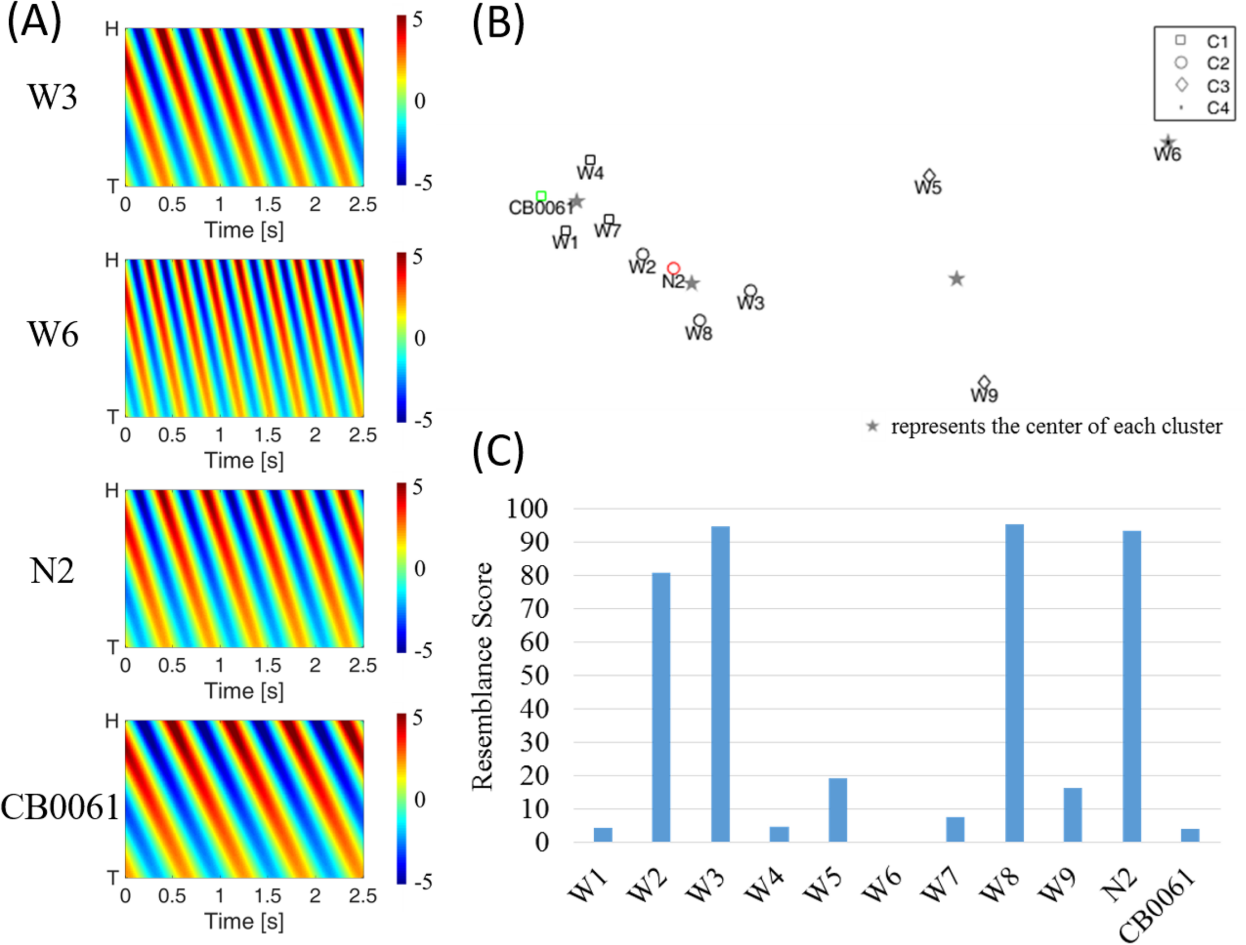
Results of simulated datasets. (A) Representative kymograms of W3, W6, N2, and CB0061. (B) K-means clustering of the measured worms according to their 12 features. (C) Resemblance scores of the simulated models against model VI.

The clustering result is shown in Fig 5B. The changes in the three parameters are reflected in the sinusoidal waveforms. When one parameter in the three models is changed each time, the parameters affected the analysis in a systematic manner. The parameters of all the simulated models (W1–W9) were changed based on N2. This comparison provided valuable information on the effect of the parameters on the distance among the data in the plot. Among the three parameters, frequency had the most influence on clustering. Therefore, the distances among W5, W6, and W7 were the largest. On the contrary, the amplitude caused lesser distance differences among W1, W2, and W3 compared with the other counterparts. The wild-type worm in model VI was obtained from the literature [36] as the reference of interest, and the resemblance scores of all the simulated models are shown in Fig 5C. The W2, W3, W8, and N2 scores were high in this comparison test, indicating their high similarity with the worm in model VI. However, CB0061, which has a dumpy phenotype, scores low because its locomotion differs from the reference worm in model VI in all aspects. A comparison of their kymograms was also consistent with the resemblance scores (Fig 5A). Results confirmed the feasibility of the gaitprint analysis method. The final resemblance score provided a reasonable prediction of the identity of the worm. Overall, the method was capable of resolving gaitprints between two worms when the difference of their curvature amplitudes, frequencies, and wavenumbers were as small as 4 pixels, 0.5 Hz, and 0.005 pixels^−1^, respectively.

### Strain recognition by the gaitprint analysis

**Fig 6:**
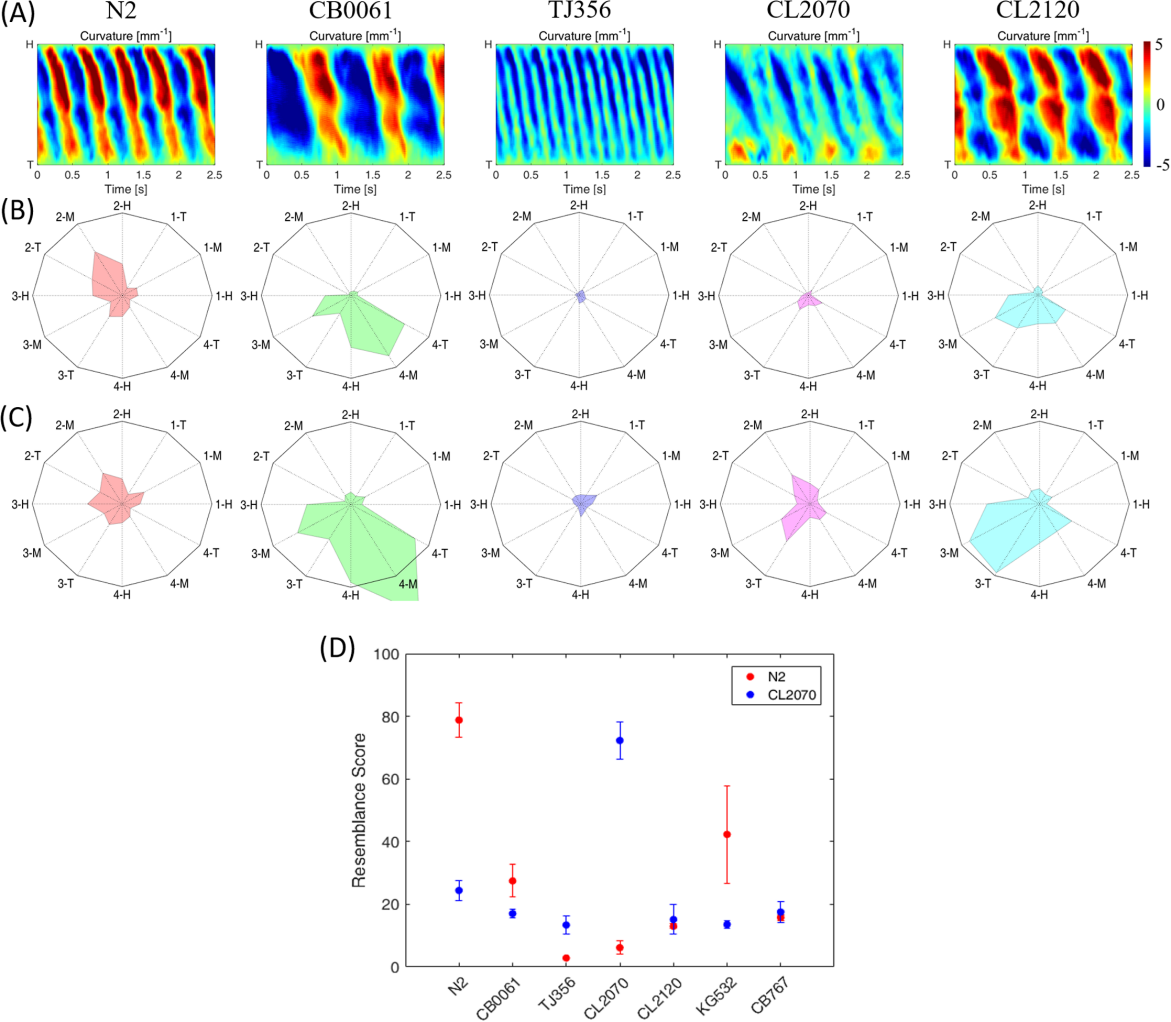
Strain recognition tests for actual worms. (A) Kymograms of the five strains, including N2, CB0061, TJ356, CL2070, and CL2120, used in the study. (B) Gaitprints of the worms against the database N2. (C) Gaitprints of the worms against the CL2070 database. (D) Resemblance scores of all the worms compared with the N2 and CL2070 databases. Red and blue circles denote comparisons achieved against the N2 and CL2070 databases, respectively.

Five worm strains were used to assess the gaitprint analysis in practice. Their kymograms derived from over 2.5 s-long videos are shown in Fig 6A. The kymograms of all the worms showed graphical differences in their swimming amplitudes, body bends, and wavenumbers. However, the hidden characteristics were further decomposed into IMFs and then analyzed according to the 12 features. Radar charts of the five strains against two selected databases, namely, N2 and CL2070, were depicted in Fig 6B and 6C, respectively.

N2 showed more traits in the second IMF because of its higher swimming frequency compared with those in the other strains. According to the gaitprints of CB0061 and CL2120, both exhibit sinusoidal swimming with strong amplitudes but low body bends (i.e., compared with N2) because their major patterns fall in the third and fourth IMFs. TJ356 and CL2070 are both rollers, and thus, they expressed low amplitudes in their radar charts. Both rollers also expressed slow head-driven locomotion (i.e., compared with N2) because of the high power generated from their front body parts. The behavior was consistent with the cues in their radar charts, because their patterns focused on the third and fourth IMFs. Notably, the radar chart of CL2070 had an amplitude of >1 because of its weak swimming amplitude (i.e., compared with N2).

After analyzing with the k-means clustering, the final resemblance scores of the measured worms against N2 and CL2120 were obtained (Fig 6D). N2 and CL2070 showed high resemblance scores (>75%) with respect to their corresponding databases. By contrast, other irrelevant worms showed low scores (<45%). In practice, a resemblance score of 100% is nearly impossible because of biological diversity. However, the resemblance score still provided a confidence level linked to the similarity between an unknown and predefined strain. For simplicity, 50% was adopted as a threshold in this study. At this threshold, the overall accuracy of the gaitprint analysis reached 89% with a total of 79 worms evaluated.

In addition to the five selected worm strains, two demonstrations were conducted to show the capability of the method. In the first case, the relationship of two additional mutant strains, namely, KG532 and CB767, with the two predefined databases were inspected without preprocessing. KG532 [*kin-2*(*ce179*) X] is a hyperactive strain with body bends higher than those of its wild-type counterpart. CB767 [*bli-3*(*e767*) I] carries the mutant *bli-3* allele, which causes blistered cuticle. Blistered cuticle is a morphological abnormality in the appearance of the worm and hinders its locomotion. The result indicated few similarities between both strains and N2 or CL2070. In particular, the resemblance score of KG532 against N2 was 42%, which was higher than those of the other cases. The increased similarity was attributed to the loss of function in the *kin-2* gene. Thus, KG532 has a subtler phenotype than other strains. In the second case, wild-type (N2) worms were cultured in two culture media, which respectively contained pure Dulbecco’s Modified Eagle’s Medium (DMEM) and DMEM mixed with Caco-2 cancer cells. Although the mechanism remains unclear, the cancerous medium indeed forms an attraction to worms according to the prior study [37]. In gaitprint analysis, the behavioral change differentiated the N2 worms in the DMEM from those in the cancerous medium. Unlike the comparisons of mutants in the first case, the little change of the behavioral phenotypes induced by the cancerous medium were unrecognized by human eyes (see S2 Movie. Note that the movie is not played in real time). By contrast, the method was sensitive to the minor locomotory changes and indicated a 53% similarity with the N2 database. Therefore, the finding indicated that the worm can be a natural biosensor to alarm the change in the composition of tested medium provided that their body languages can be interpreted. For more extended research applications, morbid worms carrying defective motor neurons or toxic proteins accumulated in muscles, such as Parkinson’s disease and Huntington’s disease, can be identified in the same manner.

## Conclusion

2D EMD has been applied in numerous signal processing applications and has been proven a powerful algorithm in data analysis. Given that the locomotory gait of *C*. *elegans* is mediated with a simple but delicate neural network, image-based locomotion can be used as an important trait to study genetic engineering, neuroscience, and strain recognition. To the best of our knowledge, this is the first study to perform image-based worm recognition through the use of 2D EMD algorithm. A gaitprint analysis method was successfully based on 2D EMD to investigate the locomotion features of *C*. *elegans*. The differences shown in the kymograms of the worms can be decomposed into four major IMFs and analyzed according to the features of the three worm body parts. Gaitprint was eventually created as a graphical map for each worm by comparing it with a predefined database. A resemblance score was then provided to facilitate the interpretation of the identity of a worm. Although a resemblance score of 100% was nearly impossible in realistic analysis because of biological diversity, a high score always linked to a high similarity between an unknown strain and a predefined database. Unknown strains can be identified without the use of the wild-type strain as reference. With an overall accuracy of more than 85%, gaitprint analysis method can provide a reasonable prediction of the identity of any unknown *C*. *elegans*. However, despite the flexibility of this method, it is only limited to worms with discernible locomotory traits. The method can be used to interpret more worm behaviors in a wide spectrum of conditions.

## Supporting information

S1 File Supporting information. (DOCX)

S1 Movie Types of locomotion. (MOV)

S2 Movie N2 worms in different buffer media. (MOV)
