# Supplementary material for: Analyzing the locomotory gaitprint of Caenorhabditis elegans on the basis of empirical mode decomposition
Source: PLoS One. 2017 Jul 24;12(7):e0181469. doi: 10.1371/journal.pone.0181469 (PMC5524362; doi:10.1371/journal.pone.0181469)
Supplement: S1 File — (DOCX) [file pone.0181469.s001.docx]

# Supporting Information

**Analyzing the Locomotory Gaitprint of *Caenorhabditis elegans* on the Basis of Empirical Mode Decomposition**

Li-Chun Lin^1^, Han-Sheng Chuang^1,2^

^1^Department of Biomedical Engineering, National Cheng Kung University, Taiwan

^2^Medical Device Innovation Center, National Cheng Kung University, Taiwan

* oswaldchuang@mail.ncku.edu.tw

## Types of locomotion.

Three types of locomotion are shown in the video (S1 Movie). The wild type worm, N2, and CB0061 belong to the first type, representing the sinusoidal phenotype. The second type include TJ356 and CL2070, which the both are genetically engineered with the *rol-6* gene and expresses a rolling phenotype. The last one is an Alzheimer model strain, CL2120, carrying human Aβ 1-42 peptides in muscles. This worm represents the uncoordinated body movement.

## N2 worms in different buffer media.

Two types of locomotion are shown in the video (S2 Movie). Wild type worms, N2, were placed in two wells of a microchips, where one of them contained pure DMEM and the other contained DMEM mixed with Caco-2 cancer cells. In the first scene, a worm is swimming in a DMEM medium. In the second scene, another worm is swimming in a mixture medium of DMEM and Caco-2.
